# Supernumerary teeth in patients with cleft lip and palate: the tooth germs do not separate

**DOI:** 10.1590/2177-6709.26.4.e21ins4

**Published:** 2021-09-10

**Authors:** Alberto CONSOLARO, Maria Carolina Malta MEDEIROS, Dario Augusto Oliveira MIRANDA, Ingrid Araújo de OLIVEIRA

**Affiliations:** 1 Universidade de São Paulo, Faculdade de Odontologia de Bauru (Bauru/SP, Brazil).; 2 Universidade de São Paulo, Faculdade de Odontologia de Ribeirão Preto (Ribeirão Preto/SP, Brazil).; 3 Oral and Maxillofacial Surgery department of the Cleft Lip and Palate Service of the “Hospital Infantil Dr. Juvêncio Matos” (São Luís/MA, Brazil).; 4 Universidade Estadual de Feira de Santana, Departamento de Saúde (BA, Brazil).

**Keywords:** Supernumerary tooth, Hyperdontia, Cleft lip and palate

## Abstract

**Introduction:**

Supernumerary teeth in cases of cleft lip and palate do not result from the division of normal germs before the formation of hard tissue. Deciduous and permanent teeth odontogenesis begins after the face has formed, either with or without the cleft.

**Discussion:**

The most acceptable hypothesis to enable understanding of the presence of supernumerary teeth on one or both sides of the cleft palate is hyperactivity of the dental lamina in its walls. This hyperactivity, with the formation of more tooth germs, must be attributed to mediators and genes related to tooth formation, under strong influence of local epigenetic factors, whose developmental environment was affected by the presence of the cleft.

**Conclusion:**

The current concepts of embryology no longer support the fusion of embryonic processes for the formation of the face, but rather the leveling of the grooves between them. All human teeth have a dual embryonic origin, as they are composed of ectoderm and mesenchyme/ectomesenchyme, but this does not make it easy for them to be duplicated to form supernumerary teeth.

## RESUMO

**Introdução:**

Os dentes extranumerários nas fissuras labiopalatinas não são resultado da divisão dos germes normais antes da formação do tecido duro. A odontogênese dos decíduos e permanentes inicia-se depois de formada a face, com ou sem fissuras.

**Discussão:**

A hipótese mais plausível para compreender a presença dos dentes extranumerários em um ou nos dois lados da fissura labiopalatina é a hiperatividade da lâmina dentária em suas paredes. Essa hiperatividade, com formação de mais germes dentários, deve ser atribuída aos mediadores e genes relacionados à formação dos dentes, sob forte influência de fatores epigenéticos locais, cujo ambiente de desenvolvimento foi afetado pela presença da fissura.

**Conclusão:**

Os conceitos atuais da embriologia não fundamentam mais a fusão de processos embrionários para a formação da face, e sim o nivelamento dos sulcos entre eles. Todos os dentes humanos têm uma dupla origem embrionária, pois se compõem de ectoderma e mesênquima/ectomesênquima, mas isso não facilita sua duplicação para formar dentes extranumerários.

**Figure 1:**
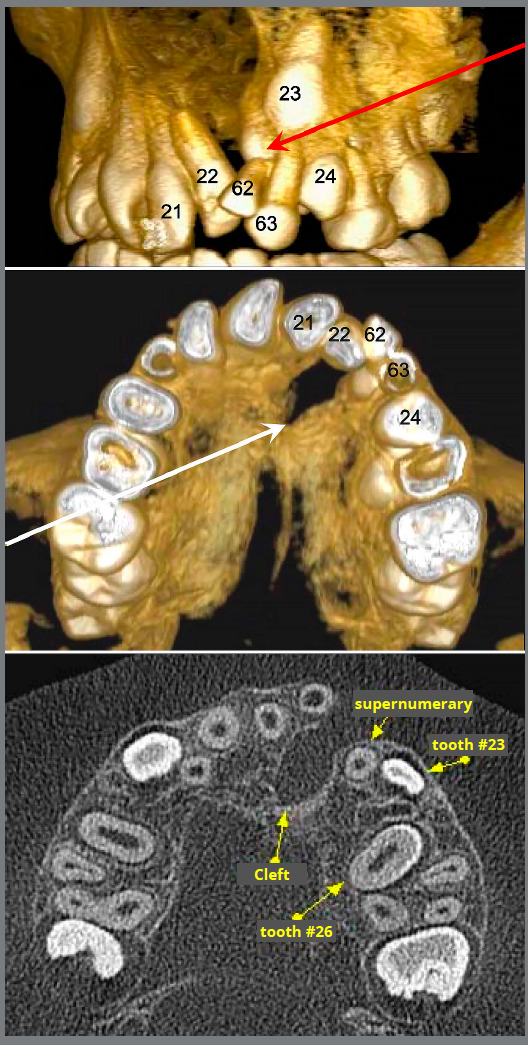
Example of a supernumerary tooth (red arrow) in front of the canine and at the margin of the cleft palate (white arrow) in tomographic images (Source: Freitas^20^, 2007).

The most frequent supernumerary teeth are the mesiodens, mandibular premolars and Bolk’s fourth molars. When they resemble the group of origin, they are denominated eumorphic supernumerary teeth, and when they have an undefined shape, they are said to be dysmorphic. In cleft lip and palate patients, the frequency of supernumerary teeth reaches up to 43.5% of cases^1^ ^-^ ^5^ (Fig 1).

## FORMATION OF THE FACE DOES NOT OCCUR BY FUSION

The formation of the face does not occur by fusion of embryonic processes, which was an older way of understanding how facial development takes place. All evidence has shown that the face is formed by leveling of the embryonic processes, except at a very specific and central point of the hard palate, from which anterior and posterior leveling is also established.^6^

**Figure 2:**
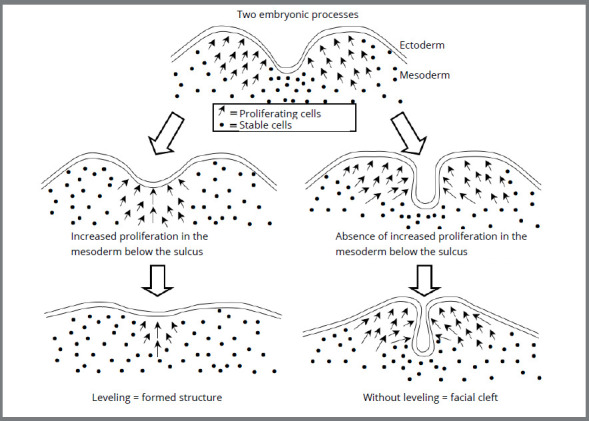
Diagram of the leveling of facial embryonic processes, on the left; and when facial cleft occurs due to lack of leveling, on the right (Source: Consolaro et al.^6^, 2017).

**Figure 3:**
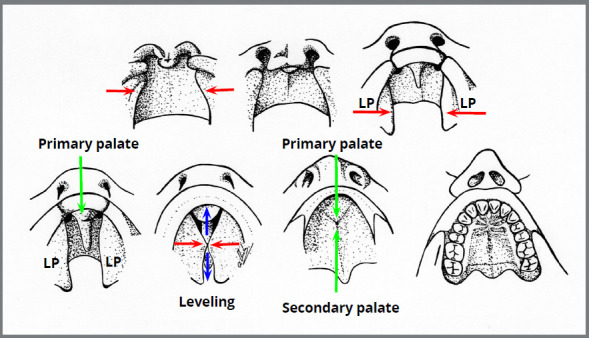
Diagram of palate formation: the two lateral palatal processes ( LP ) meet in the midline at a certain point ( red arrows ), the only point where the disappearance of the ectoderm is present, with integration or fusion of the two mesoderms. After this point, leveling ( blue arrows ) for anterior and posterior occurs, ending the secondary palate and complementing the primary palate (Source: Consolaro et al.^6^, 2017).

These concepts, of face formation mechanisms, and their evolution - from the fusion to the leveling - have been meticulously reviewed, described and presented in an article published in 2017^6^ (Figs 2 and 3).

Etiopathogenesis of some cystic diseases and lesions based on the non-fusion of processes is no longer accepted. Many of these diseases continue to be considered clinical entities, as their true etiopathogenesis has been discovered; others were diseases such as the odontogenic keratocyst, which develops in areas where fusions of embryonic processes supposedly occurred, and were not fissure lesions or cysts.^7^ ^-^ ^11^

## WHEN THE DENTAL LAMINA AND TOOTH GERMS BEGIN

The anterior part of the primitive mouth, or stomodeum, has an ectodermal lining of the external part that invaginates. This lining joins with the lining at the back of the mouth - which, in the embryo, is called the embryonic pharynx.^12^ When the buccopharyngeal membrane is broken, the two cavities join to form the final mouth. The exact site in the oral mucosa where this membrane adheres is still controversial.

The first sign of deciduous human dentition occurs around the eighth week of embryonic life, with a linear thickening in the lining and a horseshoe-shaped contour at the location of the future dental arch.^13^ This thickening will form the dental lamina that descends vertically as it enters the space that will be the mandible and maxilla. Underlying this thickening, there is an increase in the concentration of mesoderm and ectomesenchymal cells derived from the neural crest, that will later form the dental papilla and dental follicle.

This thickening of the ectodermal lining initially occurs at the region of the incisors and molars in each quadrant, which will unite, as they grow towards the anterior and posterior directions, and become equal in the region that will be the canines.

These two areas are influenced by the same mediators and tooth-related genes (Fgf-8, Pitx-2, Shh, Msx-1, Pax-9, Bmp-4), without specific differences between one and the other, as shown by Kriangkrai et al.^14^ At a certain point, the fact that these two areas have not yet become equalized or met does not mean that there was no fusion,^15^ ^,^ ^16^ ^,^ ^17^ because the face is formed by the leveling of embryonic processes by a proliferative phenomenon.

All these above-mentioned characteristics and occurrences are relative to the deciduous lateral incisor, and not the permanent lateral incisor. From the deciduous lamina, sprouts or buds will form, which will give rise to the germs of the deciduous teeth and to another lamina that arise by lingual, and will give rise to the permanent teeth - which is also called the successional lamina, and is continuous around the entire dental arch.

But there is a most important point: the first signs of formation of this successional lamina that will give rise to permanent teeth only begin to appear after the eleventh week of embryonic life.^6^ ^,^ ^13^ As mentioned before, the deciduous teeth odontogenesis begins after the eighth week, when the formation of the face and palate has been completed. The non-leveling of the processes and establishment of the cleft occurs before odontogenesis begins.

The lack of closure by leveling and the formation of the cleft represent local epigenetic factors very important in the generation of dental anomalies in the region, including the supernumerary teeth. The chronology of formation of the deciduous and permanent teeth can be retrieved in the studies conducted by Massler, Schour and Poncher,^18^ ^,^ ^19^ in 1941 and 1946, with still up to date figures, tables and illustrative diagrams.

## HYPOTHESES TO EXPLAIN SUPERNUMERARY TEETH, INCLUDING IN CASES WITH CLEFTS

The cause of supernumerary teeth is unknown. There are theories or hypotheses to explain them, highlighting the following:

1. The hyperactivity of the dental lamina, represented by excessive induction for this laminar and continuous tissue to form more tooth buds than the normal number. This can occur due to an excessive quantity of growth factors or mediators, especially in the areas of clefts, which lead to more teeth appearing than were originally programmed by the genes.
2. Atavism, or phylogenetic reversal, which represents current manifestations of distant ancestral characteristics - as a possible occurrence of third dentition, which has never been described or observed in primates and predecessors of the human race. This theory represents a theoretical and very imaginative presumption.
3. The sectioning, into two or more parts, of a tooth germ prior to the formation of hard or mineralized tooth tissue, but due to an unknown cause that has never been demonstrated *in vivo*.
4. Some cases of supernumerary teeth are familiar, but heredity is not necessarily linked to the etiopathogenesis in the majority of cases. Heredity as a factor in supernumerary teeth still needs to be further investigated, but randomness does occur in almost all cases.

## CLEFT LIP AND PALATE DO NOT OCCUR CONCOMITANTLY WITH THE BEGINNING OF ODONTOGENESIS

On the lateral walls of cleft palate, there is frequent presence of supernumeraries in the form of a extra lateral incisor on the side corresponding to the premaxilla or as a tooth similar to the lateral incisor on the maxillary side of the cleft, also identified as precanine.^1^ ^-^ ^5^ These teeth may also be dysmorphic, as they do not have the morphology of the dental group that gave rise to them, but their tissues are microscopically indistinguishable from eumorphic and normal teeth.

An explanation often given for these supernumerary teeth in clefts is that the supposed lack of fusion - an event that could never be demonstrated, even in a rudimentary way - would cleave the tooth germ into two parts, thus giving rise to two independent teeth, with one of them being supernumerary. In the formation of clefts, no cleavage occurs, and there is no external force that separates the structures, such as the maxilla and the teeth, which have already been formed. In this period, the teeth have not yet been formed.

It is difficult to imagine this occurring, as the formation of the face by leveling, or even if it was by fusion, occurs between the fourth and eighth week of embryonic life, while the first traces or tissue changes for the formation of the band and dental lamina that will give rise to permanent teeth occur from the eleventh week.

The phenomena of formation of the face and the cleft lip and palate, and the presence of tooth germs - even the germs of deciduous maxillary lateral incisors - do not occur concomitantly. In the case of permanent teeth, the formation of germs occurs much later than the occurrence of clefts, which occurs at a much earlier stage than the formation of the dental lamina of permanent teeth, called the successional lamina.

The leveling of embryonic processes means their gradual increase by proliferation of the mesoderm at the bottom of the grooves, depressions and valleys formed between them, thereby leveling the surfaces. Embryonic processes are protuberances and elevations that level out at the top of their projections (Figs 2 and 3). Hyperactivity of the dental lamina is suggested to be the explanation for the supernumerary teeth, which could be increased by the accentuated epigenetic factors in the cleft palate area.

## ALL TEETH ARE OF DUAL EMBRYONIC ORIGIN

The formation of sprouts or buds that will form tooth germs at each point corresponding to a tooth occurs by cell differentiation that is induced by mediators such as growth factors, which are peptides that have this function in an organism in the process of formation. These mediators activate odontogenesis genes in the ectoderm and mesenchyme.^14^

The lack of leveling of the medial nasal process with the maxillary process leads to the occurrence of cleft between the maxilla and premaxilla. The initial foci of this differentiation, in the incisor and molar region, as previously mentioned, continue to expand forwards and in a posterior direction, even if there is a previously established cleft.

In the region of the cleft palate, the two separate parts will continue to receive stimuli from the mediators to give rise to tooth germs. On both sides of the dental lamina and both sides of the future maxillary lateral incisor germ, the embryonic origin is the same. The topography and location of a structure does not determine its embryonic origin, but rather to which embryonic layer those cells belong. All teeth will always have an ectodermal and mesenchymal origin.

The fact that the maxillary lateral incisor is derived from the site where the medial nasal process was leveled, and the canine is derived from the area that was topographically derived from the maxillary process, does not imply that lateral incisor and canine have a distinct or different embryonic origin. The embryonic origin or nature has to do with being derived from the ectoderm, mesoderm and endoderm, and even being of ectomesenchymal origin, as in the case of all human teeth.

The fact that the deciduous maxillary lateral incisor arises from one or another facial embryonic process does not distinguish or modify its embryonic origin or its nature, especially if we consider that the mechanism of fusion of embryonic processes is not scientifically supported: There is no evidence of these fusions, obtained by means of any analysis or methodology.^6^ All evidence shows that leveling of embryonic processes takes place, not fusion. Unfortunately, many studies, although recent, have tried to explain the embryonic phenomena of the face and teeth based on the theoretical model of the fusion of processes.

Probably, the activity on each side of the cleft, or hyperactivity of the dental lamina, explains why an supernumerary maxillary lateral incisor is sometimes formed on the side of the premaxilla, and, at the same time, another supernumerary lateral incisor in the region anterior to the canine - which has, therefore, been called the pre-canine supernumerary. In many cases of cleft lip and palate there are no supernumerary teeth, and in others only one of these two types of supernumerary teeth occurs. 

## FINAL CONSIDERATIONS

The location and organization of dental buds in the dental lamina occur by induction of mediators called growth factors, which act and activate the genes of odontogenesis.^14^ Hyperactivity represented by more mediators and an increased response to them may plausibly explain the formation of supernumerary tooth buds and germs, irrespective of whether they occur in cleft areas or not.

As the times of formation of the face and the chronology of odontogenesis do not occur concomitantly, this does not allow us to affirm that the formation of a cleft lip and palate cleaves or severs the germ of the maxillary lateral incisor, in order to give rise to supernumerary teeth that are so common in these cases. On both sides of a cleft lip and palate, mediator induction continues normally and may induce supernumerary formation, by local lamina hyperactivity at these separate ends or interfaces.
